# Isolated Myocysticercosis of Pectoralis Major: A Rare Case

**DOI:** 10.7759/cureus.55320

**Published:** 2024-03-01

**Authors:** Pramatheshwara S Aradhya, Kaushik Shet, Om Prakash, Dilpreet Singh Dhillon, Jatin Chavda

**Affiliations:** 1 General Surgery, Vardhman Mahavir Medical College and Safdarjung Hospital, New Delhi, IND

**Keywords:** isolated myocysticercosis, isolated cysticercosis, taenia solium, pectoralis major, case report, cysticercosis, myocysticerosis

## Abstract

Isolated myocysticercosis is a neglected tropical disease and a rare diagnosis, with only a handful of cases being reported in the literature. It is highlighted that recently, it has not only been limited to endemic regions but also persists globally due to widespread migration from endemic regions. We present a case of isolated myocysticercosis of the right pectoralis major without neurological involvement in a non-pork-eater. High-resolution ultrasonography is an effective method of diagnosis. Anti-helmintic drugs are effective treatment options; if not responding, surgical excision is the management of choice. Ultrasound-guided excision is a better treatment modality to prevent complications.

## Introduction

Cysticercosis is a tissue parasitic infection and a neglected tropical disease caused by Cysticercus cellulosae, an encysted larval form of the organism *Taenia solium*, the pork tapeworm. The disease, considered endemic to Africa, Southeast Asia, Eastern Europe, and Mexican regions, is now a global problem due to the large number of immigrations around the world [1,2]. The disease usually affects the central nervous system as neurocysticercosis and sometimes may also extend to involve the eyes, liver, skeletal muscles, and cardiac muscles. However, isolated involvement of skeletal muscles, i.e., solitary involvement of skeletal muscles without affecting the central nervous system, is a rare phenomenon, and only a handful of cases have been reported around the world. We present a case of solitary myocysticercosis of the right pectoralis major.

## Case presentation

A 26-year-old male patient from the northern part of India, hailing from a low socioeconomic background, presented with complaints of a lump in the right chest for one year, with associated chronic mild pain in the right side of the chest aggravating on physical exercise, was able to do normal day to day chores only rarely limiting his activity. Though the patient has mixed dietary habits, he has no history of pork consumption. No lump was visible on examination. But on palpation, a deep-seated 1x1 cm lump of firm consistency was palpable in the upper lateral part of the right thorax with restricted mobility and mild tenderness. The lump was less prominent when putting the pectoral muscles into action.

Chest radiography was grossly normal. On high-resolution ultrasonography, a well-defined hypoechoic round to oval-shaped intramuscular lesion of size 9x7mm with eccentric hyperechoic foci without any internal vascularity was seen within the right pectoralis major. Diagnosis of cysticercosis was made, and further investigations were done to look for the other most common sites of the disease. Non-contrast computed tomography of the head showed no intracranial lesions, and no evidence of neurocysticercosis was found. Indirect ophthalmoscopy was performed to look for ocular cysticercosis, and no evidence of the disease was found in the eye.

**Figure 1:**
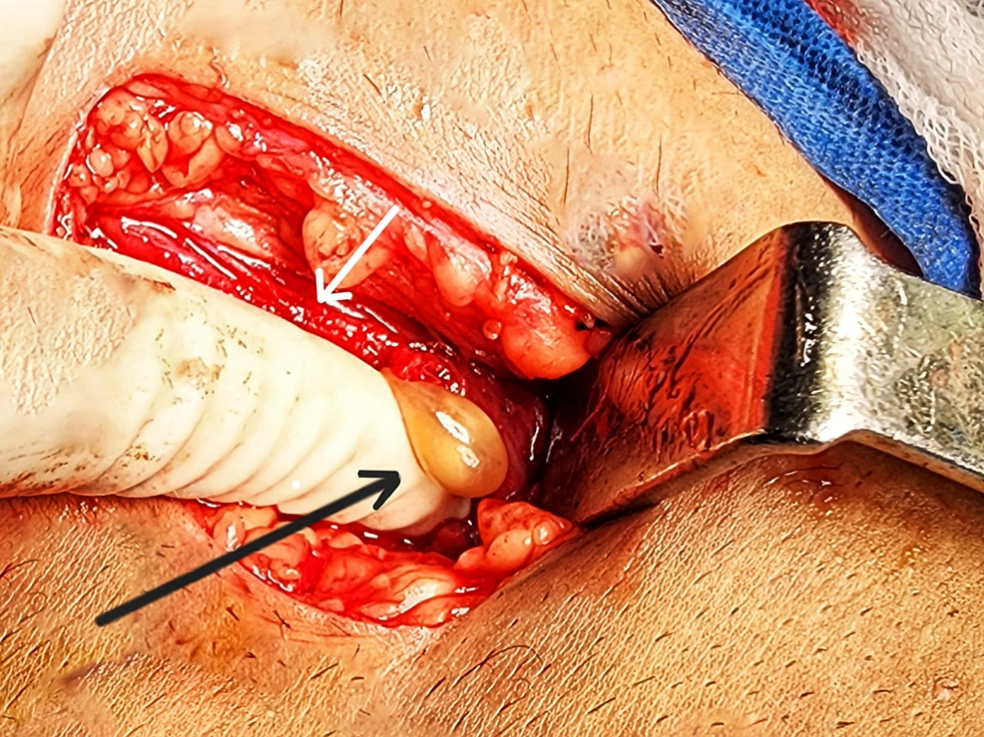
An intraoperative image of the cyst The black arrow shows the intramuscular cysticercosis; the white arrow shows the pectoralis major muscle.

**Figure 2:**
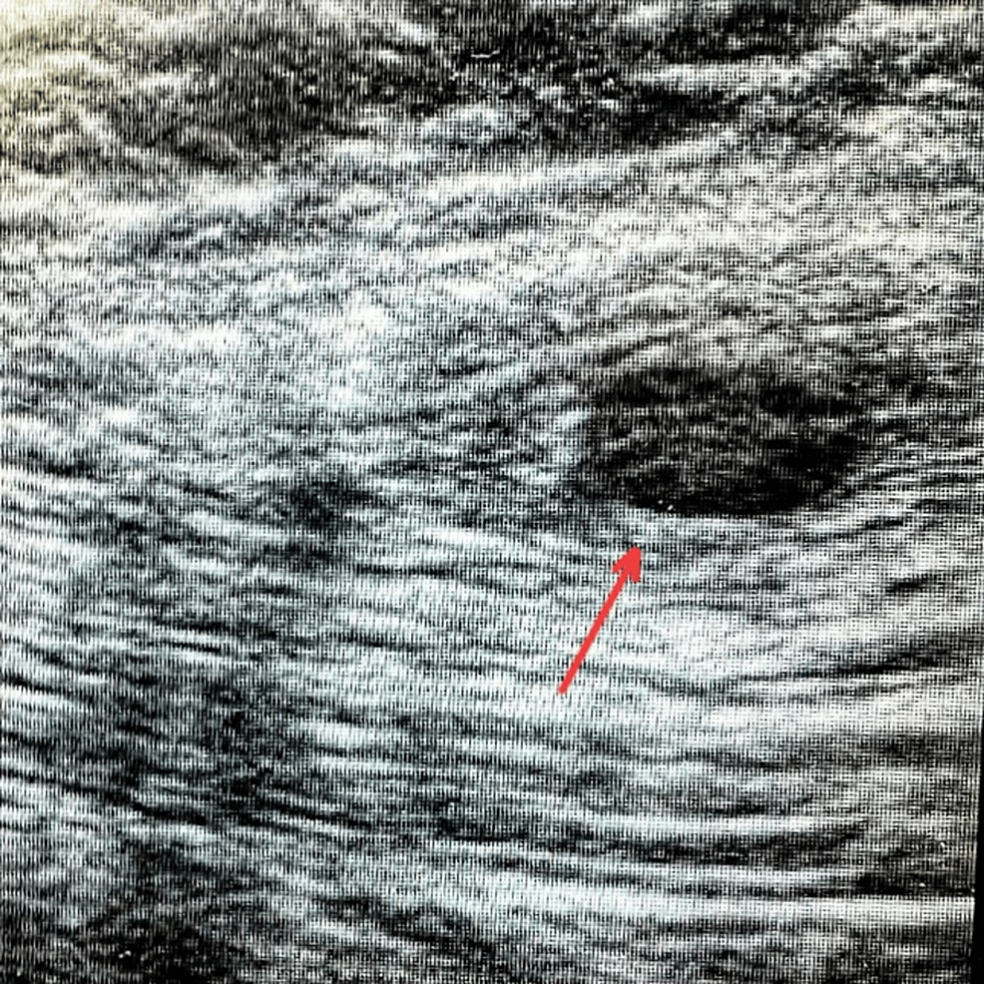
Intraoperative ultrasonography The red arrow shows a hypoechoic lesion - myocysticercosis.

**Figure 3:**
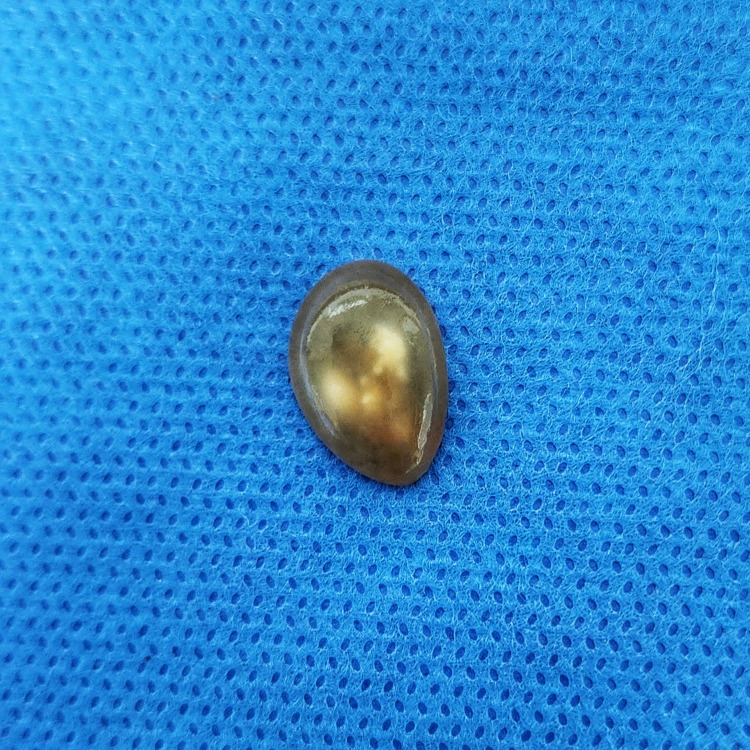
Postoperative excised cyst

The patient was treated with an albendazole tablet of 400 mg twice a day for two weeks and a diclofenac tablet of 100 mg twice a day for one week. On repeat ultrasonography, there was no decrease in the size of the cyst noted. With adequate preparation and due consent, the patient was planned for excision of the cyst under general anesthesia. Ultrasound-guided excision of the myocysticercosis from the right pectoralis major muscle was performed (Figures 1-2). In toto, excision of the cyst was done (Figure 3). The diagnosis was confirmed by demonstrating the suckers on histopathology. The patient was discharged the next day, and the postoperative period was uneventful.

## Discussion

Cysticercosis is a parasitic tissue infection caused by Cysticercus cellulosae, an encysted larval form of the parasitic organism *Taenia solium* (pork tapeworm). These infections are more commonly seen in developing tropical countries of Africa, Southeast Asia, eastern Europe, and Latin American regions. More recently, this infection has been seen to be a problem all over the world due to increased migration from endemic regions [1,2]. Though it's a widely spread disease within the subcontinent, it is under-reported [3], listing it as a neglected tropical disease.

Life cycle

*Taenia* *solium* (*T.* *solium*), being a parasite, belongs to the class of cyclophyllid cestode and has two hosts in its life cycle. For T. solium, the human is the definite host, and the pig is the intermediate host. Infection in humans occurs when they eat undercooked pork, which is lodging cysticercus cellulosae, the infective larval form of the parasite within. Once in the human digestive tract, the outer shell undergoes excystation with the help of gastric juices, following which the head of the parasite known as scolex is left behind. Scolex consists of four cup-shaped structures called suckers and a double row of hooklets known as rostellum, which facilitates the anchoring of the organism to the intestinal epithelium. Once attached, the parasite receives nutrition from the host and undergoes development and maturation over five to 12 weeks. After maturation, eggs and proglottids are released into the feces. These eggs then enter the pig through a contaminated food chain. Eggs on reaching the intestine of the pig, oncospheres are released, which then penetrate intestinal mucosa to enter mesenteric vessels and then lodge in different tissues via circulation, develop into cysts, and cause cysticercosis within the pigs [4].

With improper hygiene and sanitation practices, contamination of the water bodies can occur, including the crops and vegetables. Ingestion of this contaminated water or vegetables can cause cysticercosis in humans, even in the vegetarian population or in persons not eating pork [5]. This way, humans can also act as intermediate hosts by fecal-oral transmission due to improper food handling and sanitation. Retrograde transmission of the released proglottids back into the stomach can cause autoinfection within the same individual [4].

Pathogenesis and presentation

On entering circulation, cysts can lodge in any tissue, but most commonly, they are seen in the brain, eyes, subcutaneous tissue, and muscles. Once lodging in various tissues occurs, cysts can remain viable for a long duration since living larvae evade immune recognition. The number of cysts can vary from solitary to a few hundred [6].

The presentation of the disease varies according to the location of the cyst. Cysticercosis of the nervous system is more common than cysticercosis of other areas of the body. Cranial predominance of the cysticercosis can be attributed to high blood flow to the brain. Myocysticercosis clinically presents in three types: myalgic, myopathic, and pseudohypertrophy. The myalgic variety occurs after the death of the larvae. An immune reaction is triggered by the dead larvae, leading to an acute inflammatory response with local pain, resulting in myalgia. Myopathic type is when there is degeneration of the cyst with chronic minimal leakage of the fluid, which gives rise to chronic inflammation and mass or an abscess. The pseudohypertrophy type is when there is the formation of multilocular cysts within the muscles [7,8]. On citing an inflammatory reaction, fibrosis can occur around the cyst, which can further impinge on surrounding nerves or vessels and can cause compressive symptoms.

Diagnosis

The skiagram rarely shows any abnormality except calcified puffed rice lesions, which can be noted in the calcified cyst. High-resolution ultrasonography is a non-invasive, non-ionizing, cheaper, and easily available diagnostic modality with good sensitivity to attain the diagnosis. On ultrasonography, it can be visualized as a hypoechoic or anechoic cyst with minimal fluid or inflammatory mass around it. Sometimes, the classical scolex can be placed eccentrically within the cyst. If calcified, multiple elliptical calcifications can be seen within the soft tissue [9]. MRI is a better and gold standard investigating modality for diagnosing the disease. It appears as hypointense in T1-weighted images and hyperintense in T2-weighted images.

Fine needle aspiration cytology (FNAC) can be a rapid and useful modality. In FNAC, it presents as a collection of eosinophils, neutrophils, histiocytes, or granuloma formation. Collected hooklets or scolex confirm the diagnosis. However, the sensitivity is low since the aspirated sample may not be representative of the disease [10]. The existence of the hooklets or suckers in the histopathology after excision confirms the diagnosis.

Newer immunological modalities like enzyme-linked immunoelectrotransfer blot (EITB) are effective methods in demonstrating anti-cysticercal antibodies [11].

Prevention and treatment

Solitary involvement of the subcutaneous tissue or the muscle needs no further treatment unless they are symptomatic. Albendazole and praziquantel are the two mainstays of anti-parasitic therapy. Albendazole 10-15 mg/kg/day for two weeks and praziquantel 50 mg/kg/day for three weeks are preferred treatment options. In the presence of associated neurocysticercosis, corticosteroids should be started priorly to prevent exaggerated immune reactions and, thus, seizures. Proper investigation and treatment of intestinal taeniasis should be done to eradicate the primary source of the disease [12].

Symptomatic patients unresponsive to medical therapy can be treated with a simple excision biopsy. Ultrasound-guided excision is a better-accepted treatment modality in difficult dissection or when present near intricate structures and obviates complications.

Proper disposal of sewage and waste, good sanitary practices, hand hygiene, thorough cooking of the pork meat, and thorough washing of the fruits and vegetables when consumed raw are better personal practices effective against spreading the disease. Efforts to prevent exposure of human feces to the pigs, inspection of the meat, and good animal husbandry practices are effective measures to prevent the disease and its transmission.

## Conclusions

Isolated myocysticercosis is an uncommon disease entity, and involvement of pectoral muscle is rare. In patients presenting with complaints of intramuscular or subcutaneous mass in endemic regions or having a history of migrating from the endemic region, a high index of suspicion should be there to consider cysticercosis as a diagnosis. A vegetarian diet or nonconsumption of pork is not a criterion for exclusion of the diagnosis. High-resolution ultrasonography is an acceptable imaging modality for diagnosis. Only symptomatic patients require treatment, and surgical excision is considered only after failure of medical therapy. Guided surgical excision is a better-accepted modality of treatment.
